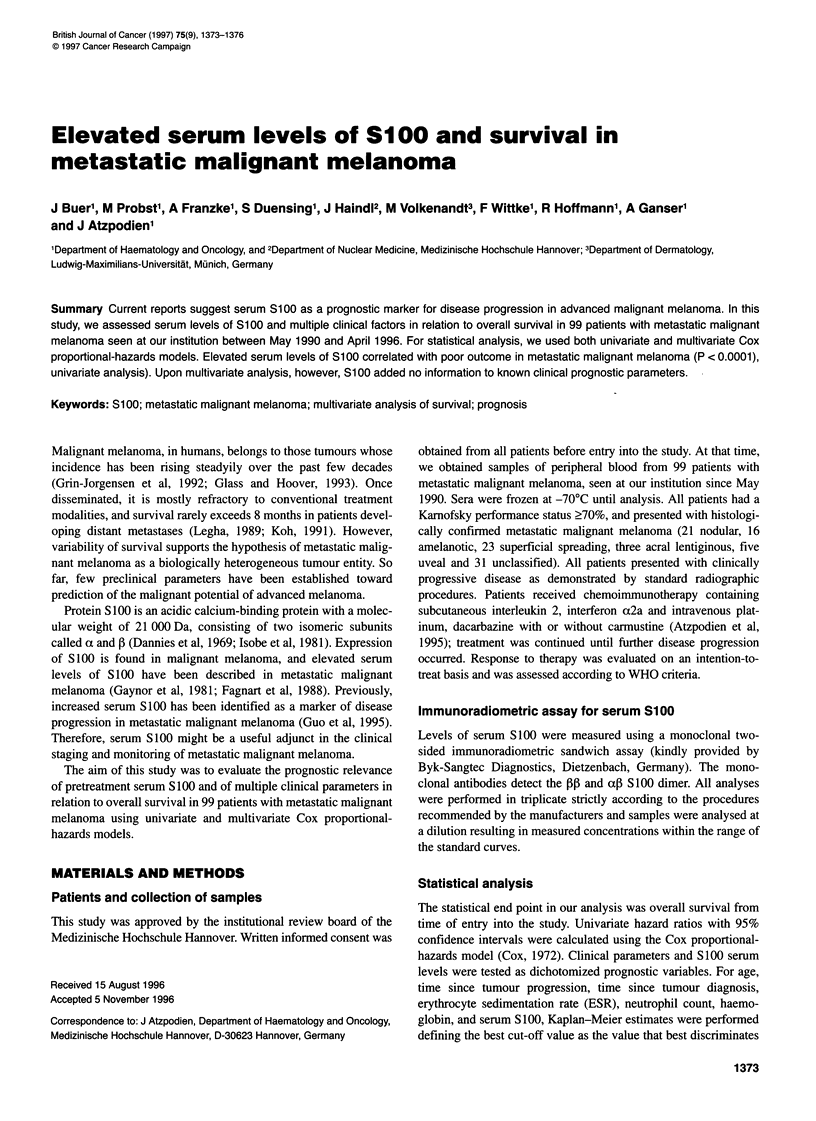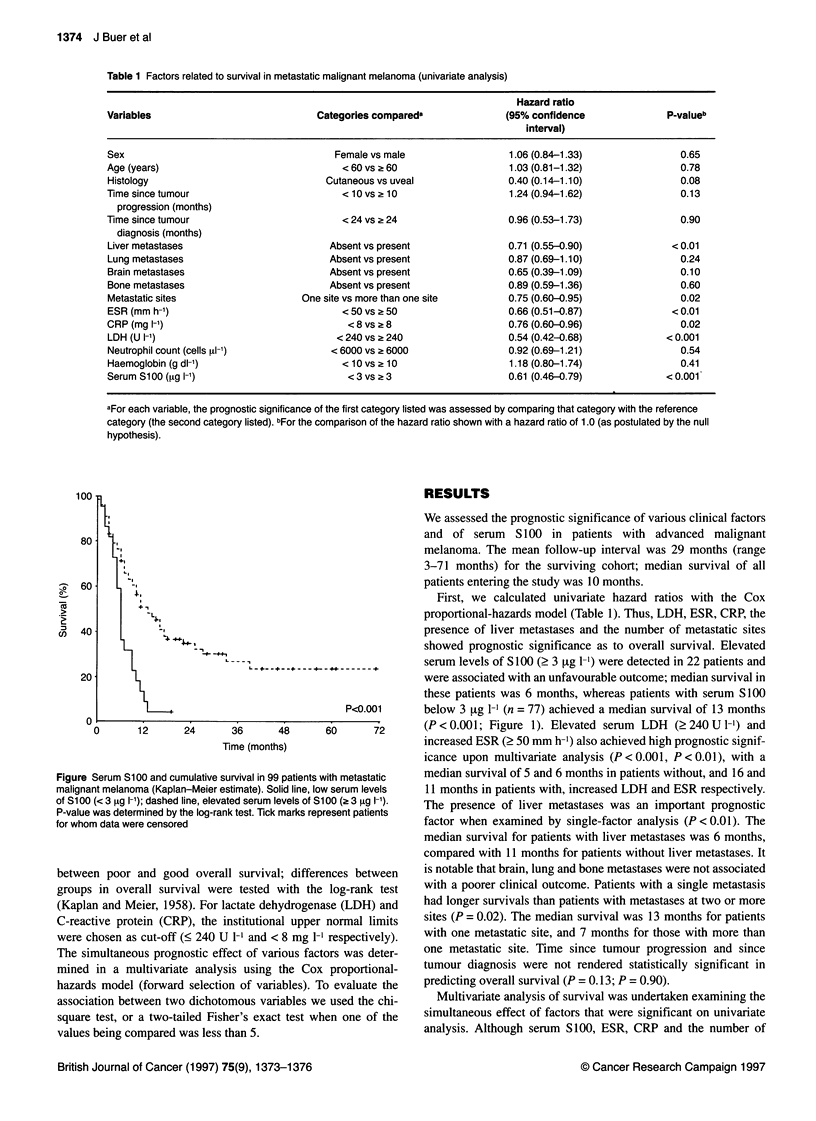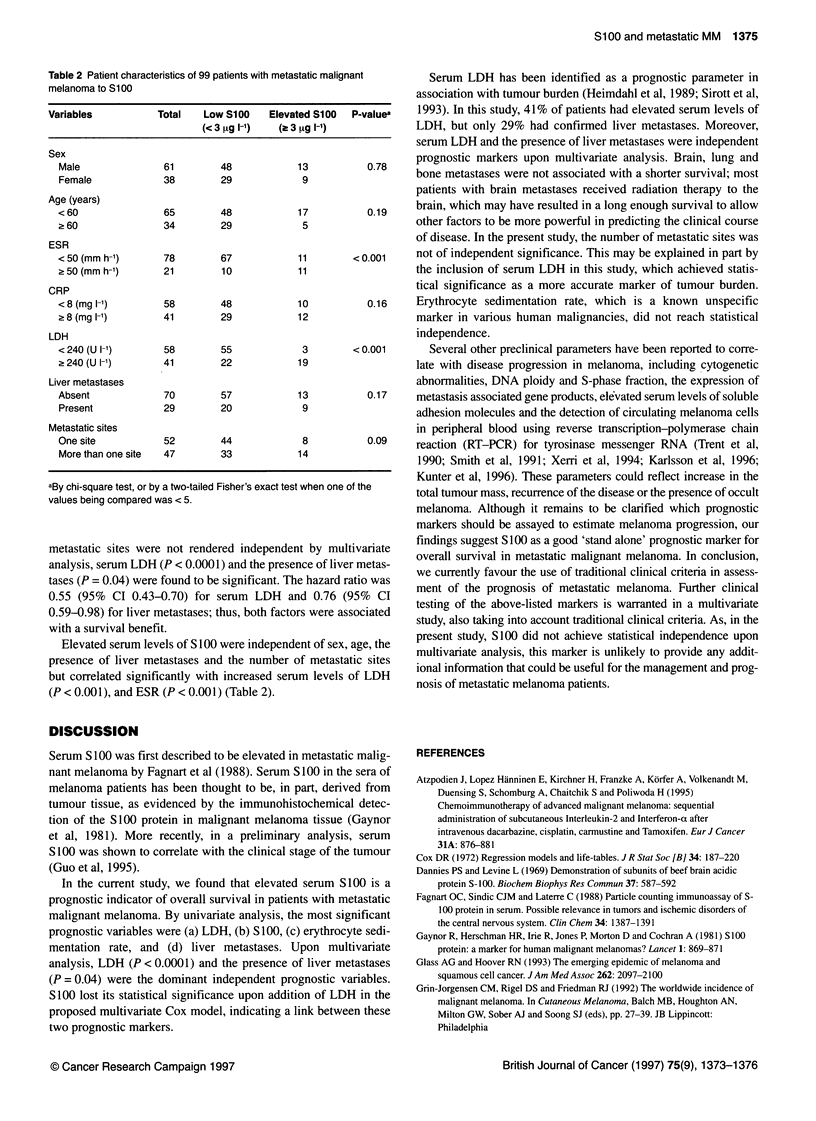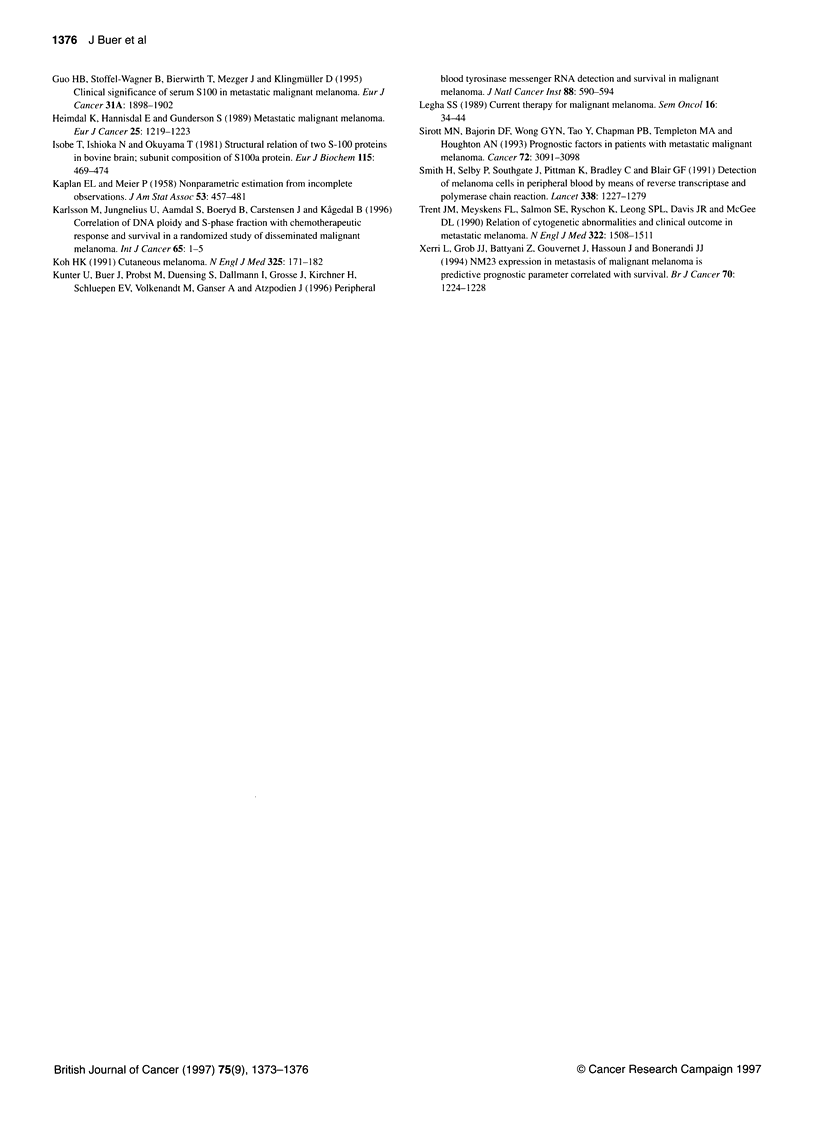# Elevated serum levels of S100 and survival in metastatic malignant melanoma.

**DOI:** 10.1038/bjc.1997.232

**Published:** 1997

**Authors:** J. Buer, M. Probst, A. Franzke, S. Duensing, J. Haindl, M. Volkenandt, F. Wittke, R. Hoffmann, A. Ganser, J. Atzpodien

**Affiliations:** Department of Haematology and Oncology, Medizinische Hochschule Hannover, Germany.

## Abstract

Current reports suggest serum S100 as a prognostic marker for disease progression in advanced malignant melanoma. In this study, we assessed serum levels of S100 and multiple clinical factors in relation to overall survival in 99 patients with metastatic malignant melanoma seen at our institution between May 1990 and April 1996. For statistical analysis, we used both univariate and multivariate Cox proportional-hazards models. Elevated serum levels of S100 correlated with poor outcome in metastatic malignant melanoma (P < 0.0001), univariate analysis). Upon multivariate analysis, however, S100 added no information to known clinical prognostic parameters.


British Journal of Cancer (1997) 75(9), 1373–1376
© 1997 Cancer Research Campaign

### Elevated serum levels of S100 and survival in metastatic malignant melanoma

J Buer[1], M Probst[1], A Franzke[1], S Duensing[1], J Haindl[2], M Volkenandt[3], F Wittke[1], R Hoffmann[1], A Ganser[1] and J Atzpodien[1]

[1]Department of Haematology and Oncology, and [2]Department of Nuclear Medicine, Medizinische Hochschule Hannover; [3]Department of Dermatology, Ludwig-Maximilians-Universität, Münich, Germany

**Summary** Current reports suggest serum S100 as a prognostic marker for disease progression in advanced malignant melanoma. In this study, we assessed serum levels of S100 and multiple clinical factors in relation to overall survival in 99 patients with metastatic malignant melanoma seen at our institution between May 1990 and April 1996. For statistical analysis, we used both univariate and multivariate Cox proportional-hazards models. Elevated serum levels of S100 correlated with poor outcome in metastatic malignant melanoma ($P < 0.0001$), univariate analysis). Upon multivariate analysis, however, S100 added no information to known clinical prognostic parameters.

**Keywords:** S100; metastatic malignant melanoma; multivariate analysis of survival; prognosis

Malignant melanoma, in humans, belongs to those tumours whose incidence has been rising steadyily over the past few decades (Grin-Jorgensen et al, 1992; Glass and Hoover, 1993). Once disseminated, it is mostly refractory to conventional treatment modalities, and survival rarely exceeds 8 months in patients developing distant metastases (Legha, 1989; Koh, 1991). However, variability of survival supports the hypothesis of metastatic malignant melanoma as a biologically heterogeneous tumour entity. So far, few preclinical parameters have been established toward prediction of the malignant potential of advanced melanoma.

Protein S100 is an acidic calcium-binding protein with a molecular weight of 21 000 Da, consisting of two isomeric subunits called α and β (Dannies et al, 1969; Isobe et al, 1981). Expression of S100 is found in malignant melanoma, and elevated serum levels of S100 have been described in metastatic malignant melanoma (Gaynor et al, 1981; Fagnart et al, 1988). Previously, increased serum S100 has been identified as a marker of disease progression in metastatic malignant melanoma (Guo et al, 1995). Therefore, serum S100 might be a useful adjunct in the clinical staging and monitoring of metastatic malignant melanoma.

The aim of this study was to evaluate the prognostic relevance of pretreatment serum S100 and of multiple clinical parameters in relation to overall survival in 99 patients with metastatic malignant melanoma using univariate and multivariate Cox proportional-hazards models.

### MATERIALS AND METHODS

### Patients and collection of samples

This study was approved by the institutional review board of the Medizinische Hochschule Hannover. Written informed consent was obtained from all patients before entry into the study. At that time, we obtained samples of peripheral blood from 99 patients with metastatic malignant melanoma, seen at our institution since May 1990. Sera were frozen at –70°C until analysis. All patients had a Karnofsky performance status ≥70%, and presented with histologically confirmed metastatic malignant melanoma (21 nodular, 16 amelanotic, 23 superficial spreading, three acral lentiginous, five uveal and 31 unclassified). All patients presented with clinically progressive disease as demonstrated by standard radiographic procedures. Patients received chemoimmunotherapy containing subcutaneous interleukin 2, interferon α2a and intravenous platinum, dacarbazine with or without carmustine (Atzpodien et al, 1995); treatment was continued until further disease progression occurred. Response to therapy was evaluated on an intention-to-treat basis and was assessed according to WHO criteria.

### Immunoradiometric assay for serum S100

Levels of serum S100 were measured using a monoclonal two-sided immunoradiometric sandwich assay (kindly provided by Byk-Sangtec Diagnostics, Dietzenbach, Germany). The monoclonal antibodies detect the ββ and αβ S100 dimer. All analyses were performed in triplicate strictly according to the procedures recommended by the manufacturers and samples were analysed at a dilution resulting in measured concentrations within the range of the standard curves.

### Statistical analysis

The statistical end point in our analysis was overall survival from time of entry into the study. Univariate hazard ratios with 95% confidence intervals were calculated using the Cox proportional-hazards model (Cox, 1972). Clinical parameters and S100 serum levels were tested as dichotomized prognostic variables. For age, time since tumour progression, time since tumour diagnosis, erythrocyte sedimentation rate (ESR), neutrophil count, haemoglobin, and serum S100, Kaplan–Meier estimates were performed defining the best cut-off value as the value that best discriminates

Received 15 August 1996
Accepted 5 November 1996

Correspondence to: J Atzpodien, Department of Haematology and Oncology, Medizinische Hochschule Hannover, D-30623 Hannover, Germany

**Table 1** Factors related to survival in metastatic malignant melanoma (univariate analysis)

| Variables | Categories compared[a] | Hazard ratio (95% confidence interval) | P-value[b] |
|---|---|---|---|
| Sex | Female vs male | 1.06 (0.84–1.33) | 0.65 |
| Age (years) | < 60 vs ≥ 60 | 1.03 (0.81–1.32) | 0.78 |
| Histology | Cutaneous vs uveal | 0.40 (0.14–1.10) | 0.08 |
| Time since tumour progression (months) | < 10 vs ≥ 10 | 1.24 (0.94–1.62) | 0.13 |
| Time since tumour diagnosis (months) | < 24 vs ≥ 24 | 0.96 (0.53–1.73) | 0.90 |
| Liver metastases | Absent vs present | 0.71 (0.55–0.90) | < 0.01 |
| Lung metastases | Absent vs present | 0.87 (0.69–1.10) | 0.24 |
| Brain metastases | Absent vs present | 0.65 (0.39–1.09) | 0.10 |
| Bone metastases | Absent vs present | 0.89 (0.59–1.36) | 0.60 |
| Metastatic sites | One site vs more than one site | 0.75 (0.60–0.95) | 0.02 |
| ESR (mm $h^{-1}$) | < 50 vs ≥ 50 | 0.66 (0.51–0.87) | < 0.01 |
| CRP (mg $l^{-1}$) | < 8 vs ≥ 8 | 0.76 (0.60–0.96) | 0.02 |
| LDH (U $l^{-1}$) | < 240 vs ≥ 240 | 0.54 (0.42–0.68) | < 0.001 |
| Neutrophil count (cells $\mu l^{-1}$) | < 6000 vs ≥ 6000 | 0.92 (0.69–1.21) | 0.54 |
| Haemoglobin (g $dl^{-1}$) | < 10 vs ≥ 10 | 1.18 (0.80–1.74) | 0.41 |
| Serum S100 ($\mu g$ $l^{-1}$) | < 3 vs ≥ 3 | 0.61 (0.46–0.79) | < 0.001 |

[a]For each variable, the prognostic significance of the first category listed was assessed by comparing that category with the reference category (the second category listed). [b]For the comparison of the hazard ratio shown with a hazard ratio of 1.0 (as postulated by the null hypothesis).

**Figure** Serum S100 and cumulative survival in 99 patients with metastatic malignant melanoma (Kaplan–Meier estimate). Solid line, low serum levels of S100 (< 3 $\mu g$ $l^{-1}$); dashed line, elevated serum levels of S100 (≥ 3 $\mu g$ $l^{-1}$). P-value was determined by the log-rank test. Tick marks represent patients for whom data were censored between poor and good overall survival; differences between groups in overall survival were tested with the log-rank test (Kaplan and Meier, 1958). For lactate dehydrogenase (LDH) and C-reactive protein (CRP), the institutional upper normal limits were chosen as cut-off (≤ 240 U $l^{-1}$ and < 8 mg $l^{-1}$ respectively). The simultaneous prognostic effect of various factors was determined in a multivariate analysis using the Cox proportional-hazards model (forward selection of variables). To evaluate the association between two dichotomous variables we used the chi-square test, or a two-tailed Fisher's exact test when one of the values being compared was less than 5.

### RESULTS

We assessed the prognostic significance of various clinical factors and of serum S100 in patients with advanced malignant melanoma. The mean follow-up interval was 29 months (range 3–71 months) for the surviving cohort; median survival of all patients entering the study was 10 months.

First, we calculated univariate hazard ratios with the Cox proportional-hazards model (Table 1). Thus, LDH, ESR, CRP, the presence of liver metastases and the number of metastatic sites showed prognostic significance as to overall survival. Elevated serum levels of S100 (≥ 3 $\mu g$ $l^{-1}$) were detected in 22 patients and were associated with an unfavourable outcome; median survival in these patients was 6 months, whereas patients with serum S100 below 3 $\mu g$ $l^{-1}$ ($n = 77$) achieved a median survival of 13 months ($P < 0.001$; Figure 1). Elevated serum LDH (≥ 240 U $l^{-1}$) and increased ESR (≥ 50 mm $h^{-1}$) also achieved high prognostic significance upon multivariate analysis ($P < 0.001$, $P < 0.01$), with a median survival of 5 and 6 months in patients without, and 16 and 11 months in patients with, increased LDH and ESR respectively. The presence of liver metastases was an important prognostic factor when examined by single-factor analysis ($P < 0.01$). The median survival for patients with liver metastases was 6 months, compared with 11 months for patients without liver metastases. It is notable that brain, lung and bone metastases were not associated with a poorer clinical outcome. Patients with a single metastasis had longer survivals than patients with metastases at two or more sites ($P = 0.02$). The median survival was 13 months for patients with one metastatic site, and 7 months for those with more than one metastatic site. Time since tumour progression and since tumour diagnosis were not rendered statistically significant in predicting overall survival ($P = 0.13$; $P = 0.90$).

Multivariate analysis of survival was undertaken examining the simultaneous effect of factors that were significant on univariate analysis. Although serum S100, ESR, CRP and the number of

© Cancer Research Campaign 1997

**Table 2** Patient characteristics of 99 patients with metastatic malignant melanoma to S100

| Variables | Total | Low S100 (< 3 μg l⁻¹) | Elevated S100 (≥ 3 μg l⁻¹) | P-value[a] |
|---|---|---|---|---|
| Sex | | | | |
| Male | 61 | 48 | 13 | 0.78 |
| Female | 38 | 29 | 9 | |
| Age (years) | | | | |
| < 60 | 65 | 48 | 17 | 0.19 |
| ≥ 60 | 34 | 29 | 5 | |
| ESR | | | | |
| < 50 (mm h⁻¹) | 78 | 67 | 11 | < 0.001 |
| ≥ 50 (mm h⁻¹) | 21 | 10 | 11 | |
| CRP | | | | |
| < 8 (mg l⁻¹) | 58 | 48 | 10 | 0.16 |
| ≥ 8 (mg l⁻¹) | 41 | 29 | 12 | |
| LDH | | | | |
| < 240 (U l⁻¹) | 58 | 55 | 3 | < 0.001 |
| ≥ 240 (U l⁻¹) | 41 | 22 | 19 | |
| Liver metastases | | | | |
| Absent | 70 | 57 | 13 | 0.17 |
| Present | 29 | 20 | 9 | |
| Metastatic sites | | | | |
| One site | 52 | 44 | 8 | 0.09 |
| More than one site | 47 | 33 | 14 | |

[a]By chi-square test, or by a two-tailed Fisher's exact test when one of the values being compared was < 5.

metastatic sites were not rendered independent by multivariate analysis, serum LDH ($P < 0.0001$) and the presence of liver metastases ($P = 0.04$) were found to be significant. The hazard ratio was 0.55 (95% CI 0.43–0.70) for serum LDH and 0.76 (95% CI 0.59–0.98) for liver metastases; thus, both factors were associated with a survival benefit.

Elevated serum levels of S100 were independent of sex, age, the presence of liver metastases and the number of metastatic sites but correlated significantly with increased serum levels of LDH ($P < 0.001$), and ESR ($P < 0.001$) (Table 2).

### DISCUSSION

Serum S100 was first described to be elevated in metastatic malignant melanoma by Fagnart et al (1988). Serum S100 in the sera of melanoma patients has been thought to be, in part, derived from tumour tissue, as evidenced by the immunohistochemical detection of the S100 protein in malignant melanoma tissue (Gaynor et al, 1981). More recently, in a preliminary analysis, serum S100 was shown to correlate with the clinical stage of the tumour (Guo et al, 1995).

In the current study, we found that elevated serum S100 is a prognostic indicator of overall survival in patients with metastatic malignant melanoma. By univariate analysis, the most significant prognostic variables were (a) LDH, (b) S100, (c) erythrocyte sedimentation rate, and (d) liver metastases. Upon multivariate analysis, LDH ($P < 0.0001$) and the presence of liver metastases ($P = 0.04$) were the dominant independent prognostic variables. S100 lost its statistical significance upon addition of LDH in the proposed multivariate Cox model, indicating a link between these two prognostic markers.

Serum LDH has been identified as a prognostic parameter in association with tumour burden (Heimdahl et al, 1989; Sirott et al, 1993). In this study, 41% of patients had elevated serum levels of LDH, but only 29% had confirmed liver metastases. Moreover, serum LDH and the presence of liver metastases were independent prognostic markers upon multivariate analysis. Brain, lung and bone metastases were not associated with a shorter survival; most patients with brain metastases received radiation therapy to the brain, which may have resulted in a long enough survival to allow other factors to be more powerful in predicting the clinical course of disease. In the present study, the number of metastatic sites was not of independent significance. This may be explained in part by the inclusion of serum LDH in this study, which achieved statistical significance as a more accurate marker of tumour burden. Erythrocyte sedimentation rate, which is a known unspecific marker in various human malignancies, did not reach statistical independence.

Several other preclinical parameters have been reported to correlate with disease progression in melanoma, including cytogenetic abnormalities, DNA ploidy and S-phase fraction, the expression of metastasis associated gene products, elevated serum levels of soluble adhesion molecules and the detection of circulating melanoma cells in peripheral blood using reverse transcription–polymerase chain reaction (RT–PCR) for tyrosinase messenger RNA (Trent et al, 1990; Smith et al, 1991; Xerri et al, 1994; Karlsson et al, 1996; Kunter et al, 1996). These parameters could reflect increase in the total tumour mass, recurrence of the disease or the presence of occult melanoma. Although it remains to be clarified which prognostic markers should be assayed to estimate melanoma progression, our findings suggest S100 as a good 'stand alone' prognostic marker for overall survival in metastatic malignant melanoma. In conclusion, we currently favour the use of traditional clinical criteria in assessment of the prognosis of metastatic melanoma. Further clinical testing of the above-listed markers is warranted in a multivariate study, also taking into account traditional clinical criteria. As, in the present study, S100 did not achieve statistical independence upon multivariate analysis, this marker is unlikely to provide any additional information that could be useful for the management and prognosis of metastatic melanoma patients.

### REFERENCES

Atzpodien J, Lopez Hänninen E, Kirchner H, Franzke A, Körfer A, Volkenandt M, Duensing S, Schomburg A, Chaitchik S and Poliwoda H (1995) Chemoimmunotherapy of advanced malignant melanoma: sequential administration of subcutaneous Interleukin-2 and Interferon-α after intravenous dacarbazine, cisplatin, carmustine and Tamoxifen. *Eur J Cancer* **31A**: 876–881

Cox DR (1972) Regression models and life-tables. *J R Stat Soc [B]* **34**: 187–220

Dannies PS and Levine L (1969) Demonstration of subunits of beef brain acidic protein S-100. *Biochem Biophys Res Commun* **37**: 587–592

Fagnart OC, Sindic CJM and Laterre C (1988) Particle counting immunoassay of S-100 protein in serum. Possible relevance in tumors and ischemic disorders of the central nervous system. *Clin Chem* **34**: 1387–1391

Gaynor R, Herschman HR, Irie R, Jones P, Morton D and Cochran A (1981) S100 protein: a marker for human malignant melanomas? *Lancet* **1**: 869–871

Glass AG and Hoover RN (1993) The emerging epidemic of melanoma and squamous cell cancer. *J Am Med Assoc* **262**: 2097–2100

Grin-Jorgensen CM, Rigel DS and Friedman RJ (1992) The worldwide incidence of malignant melanoma. In *Cutaneous Melanoma*, Balch MB, Houghton AN, Milton GW, Sober AJ and Soong SJ (eds), pp. 27–39. JB Lippincott: Philadelphia

© Cancer Research Campaign 1997

Guo HB, Stoffel-Wagner B, Bierwirth T, Mezger J and Klingmüller D (1995) Clinical significance of serum S100 in metastatic malignant melanoma. *Eur J Cancer* **31A**: 1898–1902

Heimdal K, Hannisdal E and Gunderson S (1989) Metastatic malignant melanoma. *Eur J Cancer* **25**: 1219–1223

Isobe T, Ishioka N and Okuyama T (1981) Structural relation of two S-100 proteins in bovine brain; subunit composition of S100a protein. *Eur J Biochem* **115**: 469–474

Kaplan EL and Meier P (1958) Nonparametric estimation from incomplete observations. *J Am Stat Assoc* **53**: 457–481

Karlsson M, Jungnelius U, Aamdal S, Boeryd B, Carstensen J and Kågedal B (1996) Correlation of DNA ploidy and S-phase fraction with chemotherapeutic response and survival in a randomized study of disseminated malignant melanoma. *Int J Cancer* **65**: 1–5

Koh HK (1991) Cutaneous melanoma. *N Engl J Med* **325**: 171–182

Kunter U, Buer J, Probst M, Duensing S, Dallmann I, Grosse J, Kirchner H, Schluepen EV, Volkenandt M, Ganser A and Atzpodien J (1996) Peripheral blood tyrosinase messenger RNA detection and survival in malignant melanoma. *J Natl Cancer Inst* **88**: 590–594

Legha SS (1989) Current therapy for malignant melanoma. *Sem Oncol* **16**: 34–44

Sirott MN, Bajorin DF, Wong GYN, Tao Y, Chapman PB, Templeton MA and Houghton AN (1993) Prognostic factors in patients with metastatic malignant melanoma. *Cancer* **72**: 3091–3098

Smith H, Selby P, Southgate J, Pittman K, Bradley C and Blair GF (1991) Detection of melanoma cells in peripheral blood by means of reverse transcriptase and polymerase chain reaction. *Lancet* **338**: 1227–1279

Trent JM, Meyskens FL, Salmon SE, Ryschon K, Leong SPL, Davis JR and McGee DL (1990) Relation of cytogenetic abnormalities and clinical outcome in metastatic melanoma. *N Engl J Med* **322**: 1508–1511

Xerri L, Grob JJ, Battyani Z, Gouvernet J, Hassoun J and Bonerandi JJ (1994) NM23 expression in metastasis of malignant melanoma is predictive prognostic parameter correlated with survival. *Br J Cancer* **70**: 1224–1228

© Cancer Research Campaign 1997