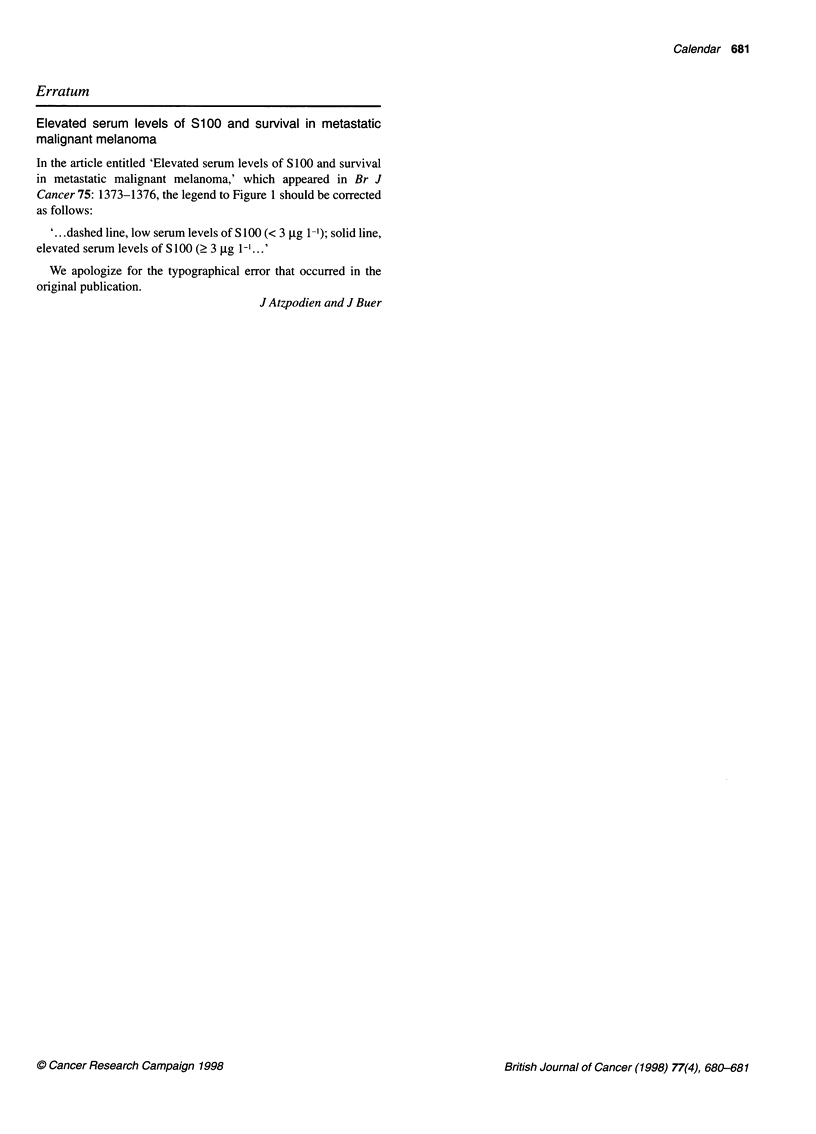# Elevated serum levels of S100 and survival in metastatic malignant melanoma

**Published:** 1998-02

**Authors:** 


### *Erratum*

### Elevated serum levels of S100 and survival in metastatic malignant melanoma

In the article entitled 'Elevated serum levels of S100 and survival in metastatic malignant melanoma,' which appeared in *Br J Cancer* **75**: 1373–1376, the legend to Figure 1 should be corrected as follows:

'...dashed line, low serum levels of S100 ($< 3\ \mu g\ l^{-1}$); solid line, elevated serum levels of S100 ($\geq 3\ \mu g\ l^{-1}$...'

We apologize for the typographical error that occurred in the original publication.

*J Atzpodien and J Buer*

© Cancer Research Campaign 1998